# Enzymology of extracellular NAD metabolism

**DOI:** 10.1007/s00018-020-03742-1

**Published:** 2021-03-23

**Authors:** Massimiliano Gasparrini, Leonardo Sorci, Nadia Raffaelli

**Affiliations:** 1 Department of Agricultural, Food and Environmental Sciences, Polytechnic University of Marche, Via Brecce Bianche, 60131 Ancona, Italy; 2 Division of Bioinformatics and Biochemistry, Department of Materials, Environmental Sciences and Urban Planning, Polytechnic University of Marche, Via Brecce Bianche, 60131 Ancona, Italy

**Keywords:** Pyridine metabolites, Ectoenzymes, Signaling proteins, NAMPT, NAPRT, NPP1

## Abstract

Extracellular NAD represents a key signaling molecule in different physiological and pathological conditions. It exerts such function both directly, through the activation of specific purinergic receptors, or indirectly, serving as substrate of ectoenzymes, such as CD73, nucleotide pyrophosphatase/phosphodiesterase 1, CD38 and its paralog CD157, and ecto ADP ribosyltransferases. By hydrolyzing NAD, these enzymes dictate extracellular NAD availability, thus regulating its direct signaling role. In addition, they can generate from NAD smaller signaling molecules, like the immunomodulator adenosine, or they can use NAD to ADP-ribosylate various extracellular proteins and membrane receptors, with significant impact on the control of immunity, inflammatory response, tumorigenesis, and other diseases. Besides, they release from NAD several pyridine metabolites that can be taken up by the cell for the intracellular regeneration of NAD itself. The extracellular environment also hosts nicotinamide phosphoribosyltransferase and nicotinic acid phosphoribosyltransferase, which inside the cell catalyze key reactions in NAD salvaging pathways. The extracellular forms of these enzymes behave as cytokines, with pro-inflammatory functions. This review summarizes the current knowledge on the extracellular NAD metabolome and describes the major biochemical properties of the enzymes involved in extracellular NAD metabolism, focusing on the contribution of their catalytic activities to the biological function. By uncovering the controversies and gaps in their characterization, further research directions are suggested, also to better exploit the great potential of these enzymes as therapeutic targets in various human diseases.

## Introduction

NAD has a well-recognized role in intracellular energetic metabolism both as a coenzyme of several dehydrogenases and as a co-substrate for enzymes controlling transcription of metabolic genes. NAD-dependent reactions are also involved in a large variety of cellular processes, including genomic stability, mitochondrial homeostasis, stress response, senescence, with profound effects on health, longevity, and age-related diseases [1–3]. Notably, also in the extracellular environment, NAD is a major signaling molecule, with a significant impact on various physiological and pathological processes [4]. In fact, once released into the extracellular space following oxidative stress, tissue injuries, and infections, extracellular NAD (eNAD) behaves as a danger signal and influences the immune system by regulating granulocytes activation and apoptosis [5, 6] and by selectively affecting the survival and suppressor function of regulators T cells [7]. Extracellular NAD also regulates the proliferation and migration of mesenchymal stem cells, as well as their immunomodulatory activity, thus contributing to maintain an optimal stem cell niche for the proper growth of hemopoietic progenitors and stem cells in the bone marrow [8]. In addition, it behaves as a neurotransmitter in enteric, peripheral, and central nervous systems [9].

Under normal physiological conditions, in mammalian serum, NAD circulates in the low micromolar range, between 0.1 and 0.5 µM; however, under inflammatory conditions, its concentration can increase up to 10  µM [10]. Several mechanisms of endogenous NAD release have been recognized, including (1) regulated efflux through Cx43 hemichannels, as reported in many cell types [11–13], (2) release from synaptic and secretory vesicles together with classical neurotransmitters [14], and (3) passive leakage across the membrane, as observed in dying cells [15].

**Fig. 1:**
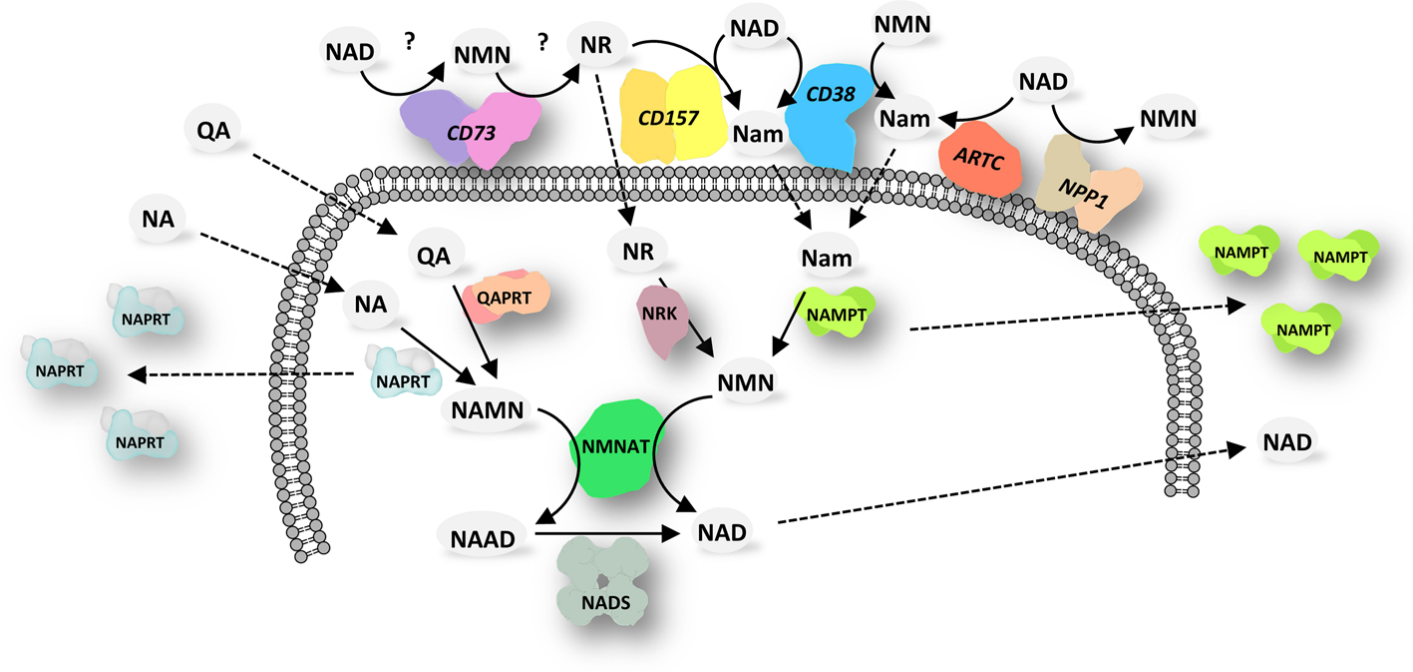
Interplay between extracellular and intracellular pyridine metabolism. Schematic overview of the extracellular pyridine metabolome and the major reactions catalyzed by NAD-metabolizing ectoenzymes. Once imported into the cell, extracellular pyridine metabolites are used to generate intracellular NAD. Abbreviations of metabolites and enzymes are described in the text. Enzymes are sketched here, and in Figs. 2 and 3, based on their biological quaternary assembly, as determined by available 3D structures. Dashed arrows indicate metabolites’  fluxes or enzymes’ secretion

Extracellular NAD can exert its signaling function both directly, by binding specific P2Y or P2X purinergic receptors [9], and indirectly, by serving as substrate for the generation of smaller signaling molecules, like the immunomodulators adenosine (ADO) and cyclic ADP ribose (cADPR), or for the covalent modification of functional extracellular proteins and receptors. Various ectoenzymes use NAD as the substrate, namely CD73, nucleotide pyrophosphatase/phosphodiesterase 1 (NPP1, also known as PC-1 and CD203a), CD38, CD157, and ecto ADP-ribosyltransferases (ARTC). With their activity these enzymes control eNAD levels, produce smaller signaling molecules, and release from eNAD various pyridine metabolites, like nicotinamide (Nam), nicotinamide riboside (NR), and nicotinamide mononucleotide (NMN), that form an extracellular pool of potential NAD precursors. Indeed, these metabolites can be taken up by the cell and used for the regeneration of intracellular NAD (Fig. 1).

Notably, two enzymes involved in intracellular NAD biosynthesis are also found in the extracellular environment. They are nicotinamide phosphoribosyltransferase (NAMPT) and nicotinic acid phosphoribosyltransferase (NAPRT) that catalyze key reactions in the intracellular NAD salvaging pathways [16]. The circulating forms of these enzymes have a cytokine-like behavior, with pro-inflammatory functions [17]. Whether they might contribute to eNAD production using the available NAD precursors has not yet been demonstrated.

Several reviews have covered in the years the multiple pathophysiological roles of the enzymes responsible of the metabolism of eNAD and its metabolites in immunomodulation, inflammation, tumorigenesis, and other diseases. However, limited attention has been paid to their catalytic properties and the contribution of their catalytic activity to the signaling function. In this review, after a description of the eNAD metabolome, we summarize the biochemical properties of the enzymes involved in eNAD metabolism.

## Extracellular pyridine metabolites

**Fig. 2:**
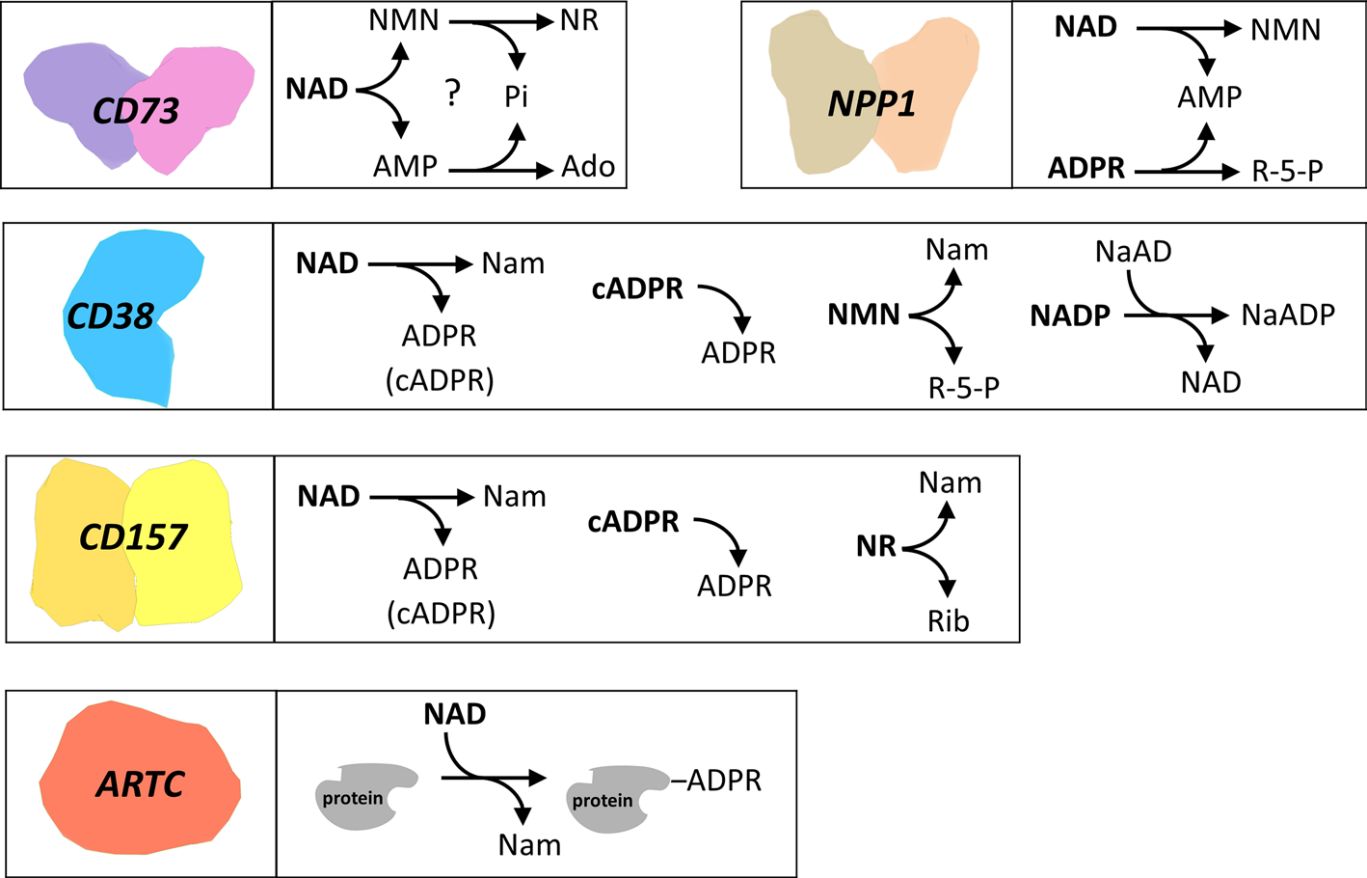
NAD metabolizing ectoenzymes. The activities of the ectoenzymes on NAD and its derivatives are shown. Abbreviations of metabolites are described in the text

Several nucleotidases are present on the mammalian cell surface that can catalyze the cleavage of eNAD, generating signaling molecules and, at the same time, releasing NAD building blocks that can be reused to maintain the intracellular levels of the coenzyme. The catalytic activities of these NAD hydrolyzing ectoenzymes are summarized in Fig. 2. In particular, ARTC, CD38, and CD157 hydrolyze the N-glycosidic bond of NAD, simultaneously transferring the ADP ribose (ADPR) moiety to specific acceptor proteins (in case of ARTC) or water (in case of CD38 and CD157), releasing Nam as the common product. CD73 and NPP1 hydrolyze the NAD pyrophosphate bond, yielding the two mononucleotides AMP and NMN. NMN can be further dephosphorylated to NR by CD73 itself, although very recently the activity of CD73 on NMN and NAD has been disputed [18].

The pyridine metabolites released by these ectoenzymes, i.e.*,* Nam, NR, and NMN, can enter the cell and can be used to regenerate NAD (Fig. 1). Nam, which has been found to be actively imported, although its transporter is still unknown [19, 20], is salvaged to NAD through the consecutive actions of the enzymes NAMPT and nicotinamide mononucleotide adenylyltransferase (NMNAT) [21]. NR can enter the cell through equilibrative nucleoside transporters [22, 23] and converted to NAD by the activities of NR kinase (NRK) and NMNAT [24]. Alternatively, in the extracellular space NR can be converted to Nam by the activity of CD157 [25]. Some studies have shown that NMN needs to be processed to NR prior to enter the cell [23, 26–28], whereas other studies support the direct uptake of the mononucleotide [29, 30]. The complexity in the detection and quantification of NR and NMN is the major reason why the mechanism of NMN transport into the cell is still under debate [28, 31], and why we still lack a clear picture of NR and NMN availability in the extracellular space. In particular, contrasting data have been provided on the levels of plasma NMN, which is either undetectable or circulating from 7 nM to about 50 µM [26, 32–35]. Nanomolar concentrations of NMN have been determined in the human cerebrospinal fluid, and both NR and NMN in the nanomolar range have been found in ascites exudates of tumor-bearing mice [22, 36], although these measurements should be taken with caution as the origin of these molecules from contaminating cells cannot be ruled out. Determination of NR, NMN, and NAD in biological fluids remains a challenging task, mainly due to limited available information on the stability of the molecules during sample handling and processing [36].

Very recently, the reduced form of NR (NRH) has been demonstrated to be an effective NAD precursor in mammalian cells and mice [37, 38]. Once inside the cell, with a mechanism yet to be identified, NRH is phosphorylated to NMNH by adenosine kinase, and the formed NMNH is adenylated to NADH by NMNAT. It is still unknown whether NRH is a physiological metabolite, but it is tempting to speculate that it might derive from the degradation of extracellular NADH, which, together with NAD, might be released by dying cells under inflammatory conditions [37].

The extracellular NAD metabolome also includes Nam and nicotinic acid (NA). In human plasma, Nam and NA, which are mostly of dietary origin, range from 0.3 to 40 µM and from 80 to 200 nM, respectively [33, 35, 39]. An additional circulating pyridine metabolite is quinolinic acid (QA), the end product in the kynurenine pathway of tryptophan catabolism. In plasma, QA circulates at low micromolar level, but it significantly increases following infections or immune challenge [40], likely deriving from cells of the immune system that accumulate substantial levels of QA upon stimulation [41]. Increased levels of QA are also found in the cerebrospinal fluid of patients with neurodegenerative disorders [42]. Both NA and QA can be taken up by cells and used to synthetize NAD after their intracellular conversion to NAMN by the enzymes NAPRT and QAPRT, respectively [21] (Fig. 1). Although NA transporters have been identified in the SLC5A8 and SLC22A13 transmembrane proteins [43, 44], the mechanism of QA release and uptake is still unknown.

The presence of deamidated pyridine nucleosides and nucleotides in extracellular fluids has been poorly investigated so far, and contrasting data on their occurrence in human plasma have been reported [35, 45]. It has been shown that human cultured cells can synthesize and release nicotinic acid riboside, which can be utilized by other cells as NAD precursor [46].

## Enzymes involved in extracellular NAD metabolism

### NAD hydrolyzing ectoenzymes

#### Ecto-5′-nucleotidase CD73

CD73 catalyzes the dephosphorylation of extracellular AMP to ADO, and represents the major control point of extracellular ADO levels [47]. Once formed, ADO binds to specific G-protein-coupled cell surface receptors and mediates diverse anti-inflammatory, angiogenic and vasoactive effects. CD73 is expressed in several tissues and cells, including immune cells like macrophages, lymphocytes, regulatory T cells and dendritic cells. It is upregulated by hypoxia and by several inflammatory mediators, and is overexpressed in several cancer types [48]. Within the tumor microenvironment, CD73-derived ADO results in tumor-driven immune suppression [49, 50] and promotes tumor angiogenesis [51, 52]. Indeed, cancer cells exploit the ADO signaling to escape from the deleterious activity of immune cells [53]. Several in vivo studies on murine models confirm a direct role of the enzyme in tumor growth and metastasization and show its potential targeting as a promising anticancer immunotherapy [54–56].

CD73 is a zinc-homodimeric protein of about 60–80 kDa, anchored to the plasma membrane at its C-terminus through a glycosylphosphatidylinositol (GPI) link. It is highly glycosylated, with glycosylation contributing about 6 kDa [47]. Whether de-glycosylation affects the catalytic activity is controversial [57, 58]. The enzyme has a broad specificity for both ribo- and deoxyribo-nucleoside 5′-monophosphates, and AMP is the best substrate with a *K*_m_ value in the low micromolar range. Maximal activity is exhibited at pH ranging from 7 to 8. It is competitively inhibited by ATP, ADP and adenosine methylene diphosphate (AMPCP), with *K*_*i*_ values in the low micromolar range. UDP, GDP, CDP, and the corresponding monophosphates, as well as concanavalin A and xanthine derivatives are also inhibitors [59, 60]. The enzyme shows some FAD pyrophosphatase activity, with a *K*_m_ for FAD similar to that for AMP, but with a 100-fold lower activity [61].

The three-dimensional structure of human CD73 has been solved in complex with various ligands, including adenosine and AMPCP, revealing an extensive active site closure movement involving its N- and C-terminal domains that would permit substrate binding and product release [62].

The high sequence and structural homology of human CD73 with *H. influenzae* NadN, an enzyme that hydrolyzes NAD to NMN and AMP, and successively NMN to NR, and AMP to ADO [63, 64], suggested that CD73 might have been endowed with the same activities [65]. Indeed, the human recombinant enzyme was found to be able to degrade NAD to NMN and AMP, and to dephosphorylate both NMN and AMP, yielding NR and ADO, respectively [64] (Fig. 2). However, the rate of ADO formation from NAD was about 160-times lower than that from AMP, raising the question whether CD73 uses NAD in vivo. Studies in cultured cells and tissues demonstrated that endogenous CD73 can sustain intracellular NAD biosynthesis by converting eNAD into eNR that enters the cell and contributes to NAD formation [22, 66]. Likewise, in endothelial cells, CD73 inhibits inflammation by modulating intracellular NAD levels through the conversion of eNMN into eNR [67]. However, the involvement of CD73 in the catabolism of eNAD and eNMN, and in the sustainment of intracellular NAD biosynthesis has been disputed in a recent study, showing that inhibition of CD73 nucleotidase activity or CRISPR/cas9-mediated knockout of the corresponding gene did not change the NAD content in a cell line model supplemented with exogenous NAD and/or NMN [18]. Furthermore, the same study could not confirm the NAD nucleotidase activity of human recombinant CD73. Further investigations are therefore warranted to indisputably assess the role of CD73 in the catabolism of eNAD.

#### Nucleotide pyrophosphatase/phosphodiesterase 1 (NPP1)

NPP1 (also known as PC-1 and CD203a) is a member of the ecto‐nucleotide pyrophosphatase/phosphodiesterase I family of enzymes. It hydrolyzes pyrophosphate- and phosphoester bonds in several nucleotide substrates, including nucleoside triphosphates and diphosphates, dinucleosides polyphosphates, 2′,3″-cyclic GAMP (cGAMP), NAD, and ADPR, releasing AMP as the common product. NNP1 is widely expressed in both lymphoid organs and nonlymphoid tissues and cells, including hepatocytes, human airway epithelial cells, the synaptic membrane of rat brain, chondrocytes, and osteoblasts [68]. A soluble form of NPP1 deriving from the intracellular processing of the membrane-bound enzyme has been identified in mouse serum and ascites fluid [69]. A major function of NPP1 is in bone mineralization and soft-tissue calcification, thanks to the generation of pyrophosphate from the hydrolysis of extracellular ATP, which functions as a negative regulator of calcification by inhibiting hydroxyapatite crystals formation [70]. Furthermore, NPP1 has been linked to insulin resistance and type 2 diabetes for its ability to interact with the insulin receptor and to inhibit the subsequent signaling [71]. The enzyme expression has been reported to be elevated in brain cancer cells, with a positive correlation between protein expression and tumor grade [72, 73]. In summary, the pathological role of NPP1 in cancer, insulin resistance, and calcification diseases has been clearly established, and the array of novel enzyme inhibitors is constantly growing [74, 75]. More recently, with the discovery that the enzyme is able to hydrolyze cGAMP, a dinucleotide with an important role in innate immunity, the involvement of NPP1 in the immune response has also been established [76]. Indeed, by lowering cGAMP levels, NPP1 affects the activation of the STING pathway, thus impairing the immune response. The concomitant generation of AMP from cGAMP can exacerbate the immunosuppressive activity of the enzyme, as AMP can be dephosphorylated to ADO by CD73.

NPP1 is a type II transmembrane glycoprotein of about 120 kDa (with glycosylation accounting for about 20 kDa), with a short N-terminal intracellular domain, a single transmembrane domain, and a large extracellular domain containing the catalytic site [47]. Disulfide bonding between the transmembrane domains mediates the protein homodimerization on the cell surface [77].

ATP is the preferred substrate among nucleoside triphosphates, with a *K*_m_ value in the low micromolar range. Lower catalytic efficiency is reported for AP_4_A and cGAMP [78]. AMP competitively inhibits the NPP1 reaction, and in vivo*,* this might prevent complete hydrolysis of substrates [79]. Optimum pH is between 9 and 10; at pH 7.4, the enzyme exhibits about 20% of its maximal activity [59].

The crystal structures of the extracellular domain of the mouse enzyme in complex with different nucleoside monophosphates, cGAMP and the nonhydrolysable 3′,3″-cyclic GAMP, have been solved, providing an explanation for the broad substrate specificity and mechanism of catalysis [80, 81]. The extracellular domain contains a nuclease-like domain, a catalytic domain with two zinc ions bound within the active site, and two somatomedin B-like domains that act as a flexible anchor linking the catalytic domain to the transmembrane region of the protein. Although catalytically inactive, the nuclease-like domain is required for catalysis and is essential for the translocation of NPP1 from the endoplasmic reticulum to the plasma membrane [82].

**Fig. 3:**
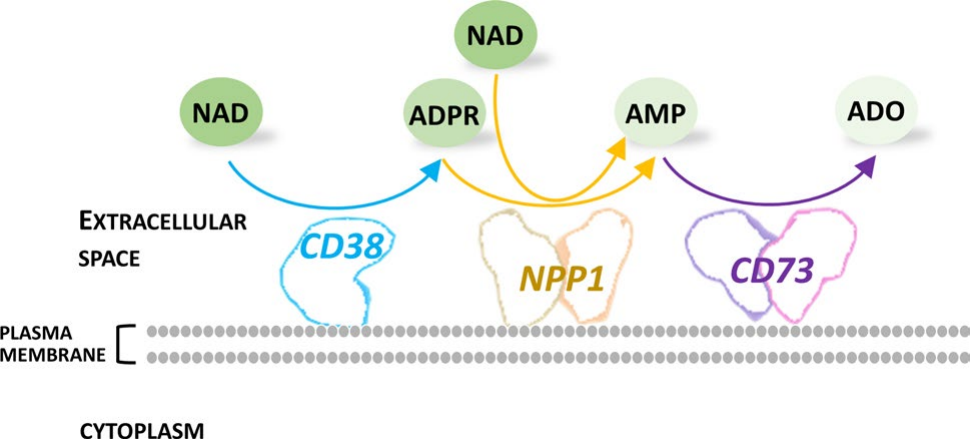
Extracellular NAD conversion to adenosine. The sequential action of the involved enzyme is shown. Abbreviations of metabolites are described in the text

The enzyme’s ability to efficiently hydrolyze NAD is described in several reports. NPP1 isolated from membrane preparations from mouse and rat liver hydrolyzes NAD at a rate significantly higher than that reported with ATP, with a *K*_m_ value in the low micromolar range [83, 84], whereas NPP1 from the plasma membrane of rat C6 glioma cells hydrolyzes NAD at a rate which is half that of ATP [85]. A partially purified preparation of enzyme from human placenta showed a *K*_m_ for NAD of 0.33 mM [86]. Furthermore, in human bronchial epithelial cells, the enzyme has been shown to metabolize also ADPR to AMP and ribose phosphate [87]. In vivo, the degradation of eNAD into AMP by NNP1 has been observed to occur in various cell types [88, 89], suggesting that the sequential action of NPP1 and CD73 can contribute to the formation of adenosine starting from extracellular NAD. In this view, NAD would be directly hydrolyzed to NMN and AMP by the NAD pyrophosphatase activity of NPP1, and AMP would be converted to ADO by CD73 (Fig. 3). On the other hand, it has been clearly established that NNP1 is involved in ADO formation from eNAD in the presence of CD38 (see below). In this pathway, NAD is hydrolyzed to Nam and ADPR by CD38, ADPR is subsequently converted to AMP by NPP1, and finally, AMP is dephosphorylated to ADO by CD73 (Fig. 3) [90, 91].

#### CD38

CD38 is both a cell surface receptor and an enzyme catalyzing the conversion of NAD into signaling metabolites, namely cADPR, ADPR, and nicotinate adenine dinucleotide phosphate (NAADP), that are all relevant Ca^2+^-mobilizers (Fig. 2). As a receptor, on the surface of immune cells, it associates with other proteins forming signaling complexes involved in the regulation of cell adhesion, differentiation, and proliferation. Its expression is upregulated after stimulation with cytokines, interferon and endotoxins, contributing to pro-inflammatory phenotypes in innate immune cells [92]. CD38 is also involved in the modulatory functions of regulatory T lymphocytes, as well as in their generation [93]. CD38-deficient mice show impairment in the humoral immune responses, regulatory T cells development, neutrophil chemotaxis, dendritic cell trafficking, and show increased susceptibility to bacterial infections [92]. In addition, CD38 has been identified as a cell-surface marker in hematologic cancers such as multiple myeloma and chronic lymphocytic leukemia and has been shown to play a role in cancer immune tolerance [94, 95].

CD38 has been characterized as type II and type III plasma membrane protein, occurring in two opposite membrane orientations, with extracellular and cytosolic catalytic site, respectively. A type III form is also present in the membranes of intracellular organelles, like nucleus, mitochondria, and lysosomes [96]. The type II is the dominant form of CD38, and the human protein is a glycoprotein of about 45 kDa, with glycosylation accounting for roughly 25% of the molecular mass [97], and dispensable for the catalytic activity [96]. CD38 also occurs in soluble form both in the cytosol and in the extracellular space [98]. Its presence is not limited to immune cells, but the protein is constitutively expressed in most tissues where it represents the major NAD consuming enzyme, significantly contributing to NAD homeostasis by affecting the availability of both extracellular and intracellular NAD and NMN [99–101]. It is, therefore, able to modulate the activity of intracellular and extracellular NAD-dependent enzymes, like sirtuins [99, 101], and ARTC [102], respectively. Its levels significantly increase in mouse tissues during aging, and this raise is the major contributor to the age-related NAD decline [99].

CD38 catalyzes the hydrolysis of the NAD glycosidic bond, releasing Nam and forming both ADPR and cADPR [103–105] (Fig. 2). ADPR is the major product, whereas only traces of cADPR are produced. A single intermediate in the active site is responsible for the hydrolysis and cyclization reactions [106]. The resolution of the crystal structure of the human enzyme in complex with NAD, ADPR, and the intermediate provided insights into the mechanism of multiple catalysis and paved the way for the design of enzyme’s inhibitors [107, 108]. In the crystal, CD38 is a monomer, which is in keeping with data suggesting that soluble CD38 exists in a catalytically active monomeric form [109], and the full-length protein can dimerize on the cell surface [110]. *K*_m_ for NAD is reported to range between 15 and 56 µM [106, 111–113], and slightly higher values have been determined for NADP (65 µM) [111] and NAADP (104 µM) [114]. CD38 also hydrolyzes NMN, with a *K*_m_ of about 149 µM and a *k*_cat,_ which is fivefold higher than that for NAD [106]. An additional substrate is cADPR (*K*_m_, 224 µM) that is hydrolyzed to ADPR, although with a rate which is only 16% that of NAD hydrolysis [111]. Optimum pH depends on the hydrolyzed substrate: NAD hydrolysis shows a broad optimum pH ranging from 6 to 8, NAADP hydrolysis is optimal between pH 4 and 5, whereas NADP hydrolysis is essentially pH independent in the range 4–8 [114, 115]. Nam and ADPR are noncompetitive and competitive inhibitors, respectively, with *K*_*i*_ values in the millimolar range [106, 111]. ATP and GTP inhibit CD38 at micromolar concentrations [108]. At acidic conditions and in the presence of suitable amounts of NA, CD38 can catalyze a nucleobase exchange reaction between NA and the Nam moiety of NADP, yielding NAADP [115]. The mechanism of NAADP formation in vivo has remained elusive until the recent finding that CD38 produces NAADP in lysosomes, using NAAD as the NA donor for the exchange reaction [116] (Fig. 2).

The products of the CD38-catalyzed reaction cADPR, NAADP, and ADPR are all intracellular second messengers targeting different calcium channels and stores [117, 118]. By modulating their levels, type III CD38 fully controls multiple calcium-dependent processes, including inflammation, insulin and oxytocin secretion, cardiogenesis, and cardiac function [119]. Recent studies have shown that the role in calcium signaling of extracellular cADPR produced on the cell surface by type II CD38 is minimal. Indeed, modulation of the ectoenzyme’s expression does not affect cellular cADPR levels [120]. On the other hand, type II CD38 significantly contributes to the generation of ADO from eNAD. In fact, on the surface of immune cells, once ADPR is produced by CD38, it is converted to AMP by NPP1, and AMP is further dephosphorylated to ADO by CD73 [91] (Fig. 3). The last step of this pathway is shared with the classical CD39/CD73 pathway that is responsible of the production of ADO from eATP. In the classical pathway CD39 converts eATP into AMP, which is then transformed to Ado by CD73. The CD38/NPP1/CD73 pathway has been shown to be operative together with the classical pathway in different populations of lymphocytes, as well as in melanoma and myeloma cells, and in different cell subsets of the bone marrow niche in multiple myeloma patients, where ADO production results in tumor-driven immune suppression [121]. Interestingly, the microvesicles released into the niche from the plasma membrane of activated or neoplastic cells express the enzymes of both pathways, being so able to produce ADO from both ATP and NAD [122]. ADO is also an important neuromodulator in the central nervous system with roles in inflammation, sleep, memory, and cognition, and several lines of evidence indicate that eNAD can be converted to ADO in cultured nervous cells, like microglia and astrocytes [123, 124].

Recent studies indicate that, besides directly sustaining the production of pro-inflammatory mediators from eNAD, the upregulation of CD38 in activated immune cells might reduce the NAD availability to pathogens; thus, limiting infections [125].

#### CD157

A paralog of CD38, likely arisen from duplication of an ancestral gene, is CD157 (also referred to as bone marrow stromal antigen BST-1), a dimeric GPI-anchored glycoprotein of about 42–50 kDa, present on the surface of several types of cells, and also detected in sera and exudates [126]. As a receptor, it regulates cell adhesion and migration, and is a marker of adverse prognosis in some types of tumors. It shares about 30% sequence identity and a high degree of structural similarity with CD38 [107, 127]. A recombinant form of human soluble CD157 displays NAD glycohydrolase and cADPR hydrolase activities, with a catalytic mechanism similar to CD38, although with an optimum pH at 4.0. In the presence of metal ions, like Zn^2+^ and Mn^2+^, the enzyme retains its maximal activity up to 6.5 [128]. Differently from CD38, CD157 is a poor catalyst. It hydrolyzes NGD, a NAD analog used to assay the enzymatic activity, with a *K*_m_ of about 610 µM, which is about 300-fold higher than the *K*_m_ value exhibited by CD38, at a rate that is about 1000-fold lower than that of the CD38-catalyzed reaction [106, 129, 130]. Even though this would suggest that the enzymatic activity of CD157 is not relevant in vivo, studies performed in mouse cultured cells show that CD157 contributes to cADPR generation, although to a lower extent than CD38 [131], and the generated cADPR has a biological effect [132, 133]. To our knowledge, the role of CD157 as an ectoenzyme in human has not been investigated.

Notably, while no hydrolysis of NR has been reported for CD38, CD157 prefers to hydrolyze NR rather than NAD, with a catalytic efficiency very close to that exhibited by CD38 towards NAD, and a *K*_m_ value for NR of about 6 nM, suggesting that it might be evolved from CD38 to bind and hydrolyze NR in vivo [25].

#### Ecto-ADP-ribosyltransferases

Protein ADP‐ribosylation is a reversible post‐translational modification that alters the function of the target protein or provides a scaffold for the recruitment of other proteins [134]. It is catalyzed by ADP ribosyltransferases (ARTs), that transfer the ADPR moiety of NAD to a specific amino acid side chain in the target protein with the concomitant release of Nam (Fig. 2). The cholera toxin-like ARTs family, named ARTC, constitutes a family of eukaryotic ARTs, structurally related to the ADP-ribosylating bacterial toxins, that catalyze arginine-specific mono-ADP-ribosylation. They are GPI-anchored to the cell membrane, or secreted into the extracellular space [135]. The family is constituted by several members with distinct tissue distribution and preferentially expressed on epithelial and inflammatory cells, such as lymphocytes and leukocytes [136, 137]. The best characterized are ARTC1 and ARTC2, the latter only found in rodents. ARTC1 is expressed predominantly in the heart, skeletal muscles, and airway. On the surface of the airway epithelial cells, it modifies the defensin human neutrophil 1, which is released following lung inflammation, thus reducing its antimicrobial and cytotoxic activities and consequently affecting the inflammatory response [138]. In the muscle cells, under basal conditions, ARTC1 ADP-ribosylates several different proteins on the cell surface and extracellular space, associated with cell adhesion, muscle contraction, and regulation of signal transduction [134]. Murine ARTC2, which exists in two allelic variants ARTC2.1 and ARTC2.2, is preferentially expressed on the surface of mature T cells [139] and is involved in the so-named NAD-induced cell death (NICD). Indeed, the low micromolar concentration of extracellular NAD released upon inflammation or tissue injury is enough for this enzyme to catalyze the ADP-ribosylation of the P2X_7_ purinergic receptor [15]. Modification of the receptor leads to its gating; thus, triggering a series of responses, finally resulting in rapid apoptotic cell death [15, 140]. This mechanism closely resembles that of bacterial ARTs, which act as killer toxins invading the mammalian host and inducing death by ADP-ribosylating host proteins [141, 142]. Regulatory T cells, which express a high level of P2X_7,_ are particularly sensitive to NICD, whereas activated effector T cells are protected from NCID due to the shedding of ARTC2 activity [143, 144]. In fact, in these cells, ADP-ribosylation of P2X_7_ results in the release of specific proteases that cleaves ARTC2 from the membrane, thus changing its targets from membrane proteins to secretory proteins [145]. These findings have established the clear role of eNAD in the homeostasis of murine T cells through ARTC2 activity [10]. In humans, the gene encoding ARTC2 is a pseudogene. Beside ARTC1 and ARTC2, three additional ARTs forms have been described: the poorly characterized ARTC5, which is a secreted enzyme, and the inactive proteins ARTC3 and ARTC4 [137].

The in vitro characterization of murine ARTC1 and variants ARTC2.1 and ARTC2.2 has been performed on the recombinant proteins expressed in eukaryotic cells and *E. coli*, respectively. ARTC1 was demonstrated to catalyze ADP-ribosylation of arginine-rich histones and to perform auto-ADP-ribosylation [146] [147]. Differences were observed in the properties of variants ARTC2.1 and ARTC2.2, depending on the species: while in mouse both variants behave as ARTC1, in rats only ARTC2.2 is able to catalyze auto-ADP-ribosylation. Evidence is reported that the auto-ADP ribosylation is multimeric, representing an ADPR polymer rather than multiple sites of mono-ADP-ribosylation [148]. In addition, while ARTC1 is endowed with a very low NAD glycohydrolase activity, such activity is readily measurable for both rat variants. In particular, a dominance of the hydrolase activity over the transferase activity has been reported for rat ART2.2 [149], with an NAD hydrolysis rate that is at least 500-fold higher than other ARTs [150].

The crystal structure of the ectodomain of rat ARTC2.2 has been solved in its apo-form and in complex with NAD, TAD, NADH and a nicotinamide analog, [150, 151], revealing a substantial structural similarity with bacterial ARTs toxins. Based on structural analyses, a catalytic mechanism has been proposed that explains how NAD hydrolysis and auto-ADP-ribosylation might occur.

### NAD biosynthetic ectoenzymes

Along with ectoenzymes that consume eNAD, the extracellular environment is inhabited by enzymes endowed with the potentiality to synthetize NAD precursors starting from circulating Nam and NA. They are NAMPT and NAPRT, which inside the cell catalyze the transfer of the phosphoribosyl moiety of PRPP to Nam and NA, generating NMN and nicotinate mononucleotide (NAMN), respectively (Fig. 1). The two reactions are key steps in the NAD salvaging routes. In particular, enzymatic studies suggest that in human, NAMPT is mainly involved in maintaining steady-state NAD levels by recycling back to the coenzyme the nicotinamide, which is generated from the intracellular consumption of NAD. On the other hand, NAPRT has been proven to be essential in boosting NAD levels under conditions of cellular stress [16]. An interesting feature of both enzymes is that they are secreted into the extracellular milieu, where they behave as cytokines, with pro-inflammatory function [152]. The mechanism of their secretion and their physiological function in the extracellular space is still uncertain. Whether they might contribute to eNAD biosynthesis is still unknown, although the absence of detectable levels of the PRPP substrate in plasma seems to rule out the in situ catalyzed formation of NMN and NAMN [33]. In addition, no data exist on the extracellular occurrence of the enzymes that convert the mononucleotides into NAD, i.e., NMNAT and NAD synthetase. The finding that eNAD concentration raises under inflammatory conditions, which is in keeping with its role as a danger signal, suggests that it likely derives from the lysis of dying cell. In these conditions, as dying cells also release their intracellular pool of ATP and PRPP, the NAD biosynthetic activities of extracellular NAMPT and NAPRT would become relevant, further enhancing eNAD levels, thus contributing to the modulation of the inflammatory response [153].

#### Nicotinamide phosphoribosyltransferase

Extracellular NAMPT (also known as visfatin/pre-B cell enhancing factor, PBEF) is secreted by various cell types, including neutrophils, microglia, macrophages, and adipocytes. The release of the enzyme is induced by cellular stress, nutritional cues, and inflammatory signals, and once secreted, the protein triggers various intracellular signaling pathways on a variety of cell types, including immune cells, adipocytes, and cancer cells [154]. In general, eNAMPT is endowed with proliferative, anti-apoptotic, pro-inflammatory, pro-angiogenic, and metastatic properties. Accordingly, circulating eNAMPT levels are frequently increased in patients with acute or chronic inflammation [155].

The molecular mechanism of eNAMPT signaling is still unknown. Although the extracellular protein is enzymatically active [156], some studies show that the cytokine-like function of eNAMPT is independent of the enzymatic activity [157]. In vitro and in vivo experiments have demonstrated the ability of the protein to directly bind Toll-like receptor 4 (TLR4) [152, 158] and C–C chemokine receptor type 5 [159, 160], which might explain how the protein activates the inflammatory response.

The secreted form of the enzyme is shown to be able to induce intracellular NAD biosynthesis in several mice tissues with consequent health benefits, including lifespan extension and maintenance of the hypothalamic function [161, 162]. The hypothesis is that eNAMPT might catalyze NMN formation directly in the extracellular space, thus supplying cells with this NAD precursor. This would imply the presence of suitable extracellular levels of the enzyme’s substrates to support the catalytic activity. However, while Nam is present in the extracellular space, PRPP is undetectable [33]. The recent evidence that eNAMPT is carried in extracellular vesicles suggests that it might enhance NMN and hence NAD biosynthesis upon internalization in the target tissue [162, 163]. This finding also discloses a possible mechanism of NAMPT secretion through exosomes and microvesicles, which is in keeping with the lack of cytokine-specific secretion sequences in the protein, and the inability of typical inhibitors of Golgi-dependent protein secretion to inhibit NAMPT secretion.

The significant duality in eNAMPT function between the pro-inflammatory effects on the one hand and the protective effects on the other has been recently ascribed to a shift of the circulating protein from a monomeric and catalytically inactive form to a dimeric active form [34]. In particular, at the physiological circulating concentration (1 ng/ml), eNAMPT behaves as an active dimeric enzyme, whereas at higher concentrations, as observed in the serum of type 2 diabetes (about 5 ng/ml), the protein is mainly in the inactive monomeric form. Notably, the monomer has pro-inflammatory effects and mediates pancreatic beta-cell dysfunction, whereas the dimer enhances beta-cell function via NAD-dependent mechanisms [34].

An additional molecular difference between intracellular and extracellular NAMPT is the acetylation extent, and deacetylation of the intracellular enzyme by SIRT1 and SIRT6 seems to control the enzyme’s secretion [161, 164].

#### Nicotinate phosphoribosyltransferase

The extracellular form of NAPRT has been characterized very poorly. It circulates in human plasma at concentrations similar to eNAMPT, in the range 1.5–2 ng/ml, but its levels significantly increase in acute inflammatory diseases, like sepsis and septic shock [152]. In septic patients, a significant association between high levels of circulating NAPRT and mortality has been observed, suggesting that eNAPRT might be a novel risk factor for sepsis. Experiments performed in human cultured cells showed that eNAPRT induces an inflammatory response in macrophages and triggers their differentiation from circulating monocytes. These effects have also been reported for eNAMPT and, in both cases, are independent of the enzymes’ catalytic activity, but rely on the binding of the proteins to the TLR4 receptor [152]. Such a common mechanism of action can be likely explained by the high degree of structural similarity between the human proteins [165]. Interestingly, bacterial NAMPT and NAPRT, although sharing with the mammalian counterparts a very similar overall architecture, are not able to elicit an inflammatory response and, accordingly, are not TLR4 ligands. This finding might be exploited to identify the structural determinants responsible for the interaction of the two human proteins with the receptor, with the ultimate aim to interfere with their inflammatory function.

## Conclusions

There is an arsenal of NAD metabolizing enzymes in the extracellular space and on the cell membrane that act as enzymes, receptors, and cytokines, with a significant impact on intracellular signaling pathways. Among them, CD38, CD157, and ARTC are enzymes that use NAD as the preferred substrate, and the involvement of their catalytic activities in various biological processes, such as immunomodulation and inflammation, has been clearly established. On the other hand, CD73 and NPP1, which do not use NAD as the preferred substrate, remain less characterized in their NAD hydrolyzing activity, and further studies are needed to investigate their involvement in the generation of ADO from eNAD in vivo. By catalyzing their reactions, the NAD hydrolyzing enzymes release small pyridine metabolites, like Nam, NMN, and NR that can sustain intracellular NAD biosynthesis, as demonstrated by several studies. However, how these metabolites, as well as those exogenously administered, are able to maintain or even increase intracellular NAD levels is still a matter of investigation. A recent work has clearly established that studies on pyridine supplementation and uptake preformed on cultured cells can be severely affected by the culture conditions, since the serum used in the culture medium, even in a heat-inactivated form, contains enzymes responsible of an efficient degradation of NAD and its intermediates [27]. Taken into consideration the presence of such activities will allow to get a clearer picture of eNAD metabolism.

Interestingly, the intracellular NAD biosynthetic enzymes NAMPT and NAPRT can be secreted into the extracellular space where they exert a pro-inflammatory function. We have still very little information on the molecular and catalytic properties of the extracellular proteins and the possible differences with the intracellular counterparts. Likewise, our knowledge of the amount of substrates and effectors of the catalyzed reactions in the extracellular space is still very limited to establish whether they might contribute to eNAD homeostasis. Similarly, whether their catalytic activity is responsible for the cytokine-like behavior is still an open question.

In conclusion, by uncovering the controversies and gaps in the enzymology of eNAD metabolism, this review suggests further research directions to better define the physiological and pathological roles of the enzymes, and to better exploit their great potential as therapeutic targets in various human diseases.
